# Regulation of fibroblast growth factor 15/19 and 21 on metabolism: in the fed or fasted state

**DOI:** 10.1186/s12967-016-0821-0

**Published:** 2016-03-01

**Authors:** Dandan Guan, Lidan Zhao, Daiwen Chen, Bing Yu, Jie Yu

**Affiliations:** Animal Nutrition Institute, Sichuan Agricultural University, No. 211 Huimin Road, Wenjiang District, Chengdu, 611130 China; Key Laboratory for Animal Disease-Resistance Nutrition of China Ministry of Education, Sichuan Agricultural University, Chengdu, 611130 China; Department of Animal and Poultry Sciences, Virginia Polytechnic Institute and State University, Blacksburg, VA 24061 USA

**Keywords:** Fibroblast growth factor 15/19, Fibroblast growth factor 21, Fed, Fast, Metabolism

## Abstract

Fibroblast growth factor (FGF) 15/19 and FGF21 are two atypical members of FGF19 subfamily that function as hormones. Exogenous FGF15/19 and FGF21 have pharmacological effects, and endogenous FGF15/19 and FGF21 play vital roles in the maintenance of energy homeostasis. Recent reports have expanded the effects of FGF15/19 and FGF21 on carbohydrate and lipid metabolism. However, the regulations of FGF15/19 and FGF21 on metabolism are different. FGF15/19 is mainly secreted from the small intestine in response to feeding, and FGF21 is secreted from the liver in response to extended fasting and from the liver and adipose tissue in response to feeding. In this work, we reviewed the regulatory effects of FGF15/19 and FGF21 on metabolism in the fast and fed states. This information may provide some insight into the metabolic regulation of FGF15/19 and FGF21 in different physiological condition.

## Background

Fibroblast growth factors (FGFs) are a group of structurally related polypeptides, involved in various biological processes such as neuronal functions, development, differentiation, and metabolism [1–3]. There have been 22 FGFs, FGF1–FGF23, identified in mouse and/or human, among which human FGF19 is the orthologous gene of mouse FGF15. The FGFs can be divided into seven subfamilies according to their gene locus, phylogenetic analyses and action modes [4].

These mammalian subfamilies are also classified into three groups based on their action mechanisms, including the intracellular FGFs, the hormone-like FGFs and the canonical FGFs [5, 6] (Table 1). The intracellular subfamily, function as nonsecreted signaling molecules and mainly plays a role in neuronal functions [7, 8]. The hormone-like subfamily, functions over long distances in an endocrine manner and mainly plays a role in metabolism [6]. Canonical FGFs, function as autocrine and/or paracrine in multiple developmental processes [2, 9, 10]. Most FGFs have a high affinity for heparin sulfate in the extracellular matrix except the endocrine FGFs, which include FGF15/19, FGF21, and FGF23, have little or no affinity for heparin sulfate [11].

**Table 1:** FGFs super family

FGFs	
Intracellular subfamily	FGF11, FGF12, FGF13, FGF14
Endocrine subfamily	FGF15/19, FGF21, FGF23
Canonical subfamily	FGF1/2/5, FGF3/4/6, FGF7/10/22, FGF8/17/18, FGF9/16/20

Fibroblast growth factors (FGFs) are a group of proteins. Currently, the FGF family consists of 22 members that can be classified into three groups and can also be divided into 7 subfamilies

FGF15/19 is mostly secreted from the small intestine in response to feeding. The expression of FGF21 is induced in multiple organs in response to diverse nutrition stressors, such as fasting and amino acid deprivation [12]. FGF15/19 is secreted from the ileum in response to feeding, it acts as endocrine hormones and takes part in the regulation of glucose and lipid metabolism [13]. After entering the portal venous circulation, FGF15/19 represses bile acid synthesis and gluconeogenesis, promotes glycogen synthesis [14], and stimulates gallbladder filling [15]. Unlike other members in the FGF family, FGF21 is a newly discovered factor for metabolism [5], it lacks heparin-binding domain, and has no effect on promoting mitosis and proliferative activity [16, 17]. In response to fasting, FGF21 expression is induced in the liver [18, 19]. Secreted FGF21 acts as an endocrine hormone to induce ketogenesis and gluconeogenesis. In response to feeding, FGF21 expression is induced in WAT and liver [20–23]. In WAT, FGF21 acts through an autocrine mechanism to stimulate PPARγ activity and glucose uptake, and via an endocrine mechanism to repress lipolysis in liver [14, 18, 24]. Therefore, we reviewed the regulatory effects of FGF15/19 and FGF21 on nutrient metabolism in the fast and fed states in the present work.

### Receptors of FGF15/19 and FGF21

FGFs exert their function by binding to their tyrosine kinase receptors, FGF receptors (FGFRs). FGF receptors consist of three extracellular immunoglogulin (Ig)-like domains and a single transmembrane domain [25]. Four FGF receptors, FGFR-1 through FGFR-4, have been identified so far [26]. There are many types of FGFs, which require the diversity of FGFR. However, by alternated splicing, the same FGFR genes could generate a variety of different isoforms [27]. The most variant parts are the extracellular Ig domains. FGFR may lack one Ig domain or use different exon for the same Ig-like domains. There are three types of third Ig domains (IIIa, IIIb, and IIIc). The binding of FGF to its receptor requires proteoglycans, namely heparin or heparin sulfate [28], which protects FGF from degradation and creates a local reservoir of FGF.

**Fig. 1:**
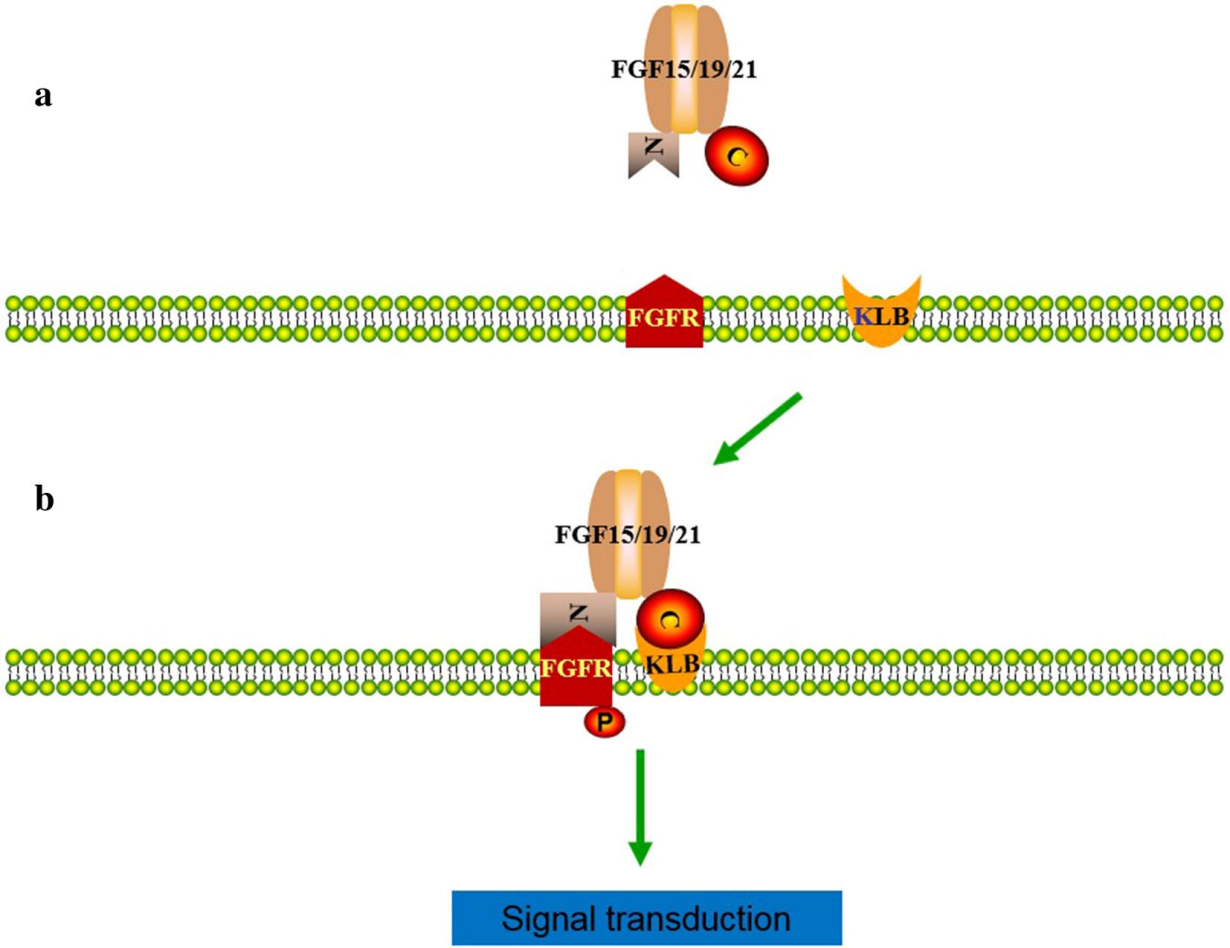
The mechanism of FGF15/19 and FGF21 receptor activation. **a** FGFR and KLB that are constitutively associated on the plasma membrane comprise the FGF15/19 and FGF21 receptor, but the receptor is silent without FGF15/19 and FGF21. **b** Once FGF15/19 and FGF21 come into the vicinity of the receptor, it associates with its receptor. FGF15/19 and FGF21 through its C-terminus bind to KLB, and via its N-terminal part to contact FGFR. Binding of FGF15/19 and FGF21 to FGFR and KLB triggers the receptor phosphorylation, followed by downstream signal transduction and cellular functional responses

In addition to FGFRs, the FGF19 subfamily members need Klotho (KL) or βKlotho (KLB) protein as a co-receptor to activate their signaling pathway [3] (Fig. 1). KL is a single-pass trans-membrane protein, which has two homologous domains in the extracellular domain and a short intracellular tail [29]. KLB is expressed in liver, adipose tissue, pancreas and muscle, whereas KL is expressed in kidney and intestine [30]. KL was first identified in mice as an age suppressor gene. A defect in KL resulted in multiple ageing-like phenotypes, and KL overexpression extends life span in mice [31]. The study with global KLB-knockout mice showed that KLB is essential for most of the physiological functions of FGF15/19 and FGF21 [30].

FGF19 has low affinity for heparin. KLB is essential for FGF15/19 interaction with FGFRs 1c, 2c, 3c and 4c [30, 32]. KLB appears to stabilize the interaction of this ligand with its receptor, perhaps acting as a surrogate for heparin [33]. FGF15/19 is also able to interact directly with FGFR4 in the absence of KLB in a heparin-dependent manner [32, 34]. Therefore, FGF19 can activate FGFR4 in a KLB-dependent or heparin-dependent manner [33]. A recent study noted that FGF15/19 binds both FGFR1 and FGFR4 in the presence of KLB with comparable affinity, but not to FGFR1 alone although there is 10 % binding to FGFR4 alone. Like FGF15/19, FGF21 binds to KLB in complex with FGFR1c, 2c, or 3c. FGF21 has much higher affinity to FGFR1 than FGFR4 in the presence of KLB [29].

It has been believed that FGF21 forms complexes with FGFR and KLB to activate downstream signaling pathways [35–37]. However, in vitro experiments show that KLB is indispensable for FGF21 [35, 36]. What causes these controversial results is still unclear. Possible explanations proposed by researches were the specific characteristics of cultured cells and an artificial abundance of KLB or FGF21 by adding them into the medium [38].

Typically, FGF binding to FGFR requires heparin sulfate cofactor that limits the diffusion of FGFs from their site of release, so FGF acts as a paracrine or autocrine factor [39]. However, FGF19 subfamily members have low affinity to heparin sulfate, which allow them to enter the circulation and function as hormones [16]. They have several effects that are similar to those of insulin, including stimulation of glycogen synthesis and suppression of gluconeogenesis [13].

### Role of FGF15/19 and FGF21 in metabolism

#### Regulation of FGF15/19 on metabolism

**Fig. 2:**
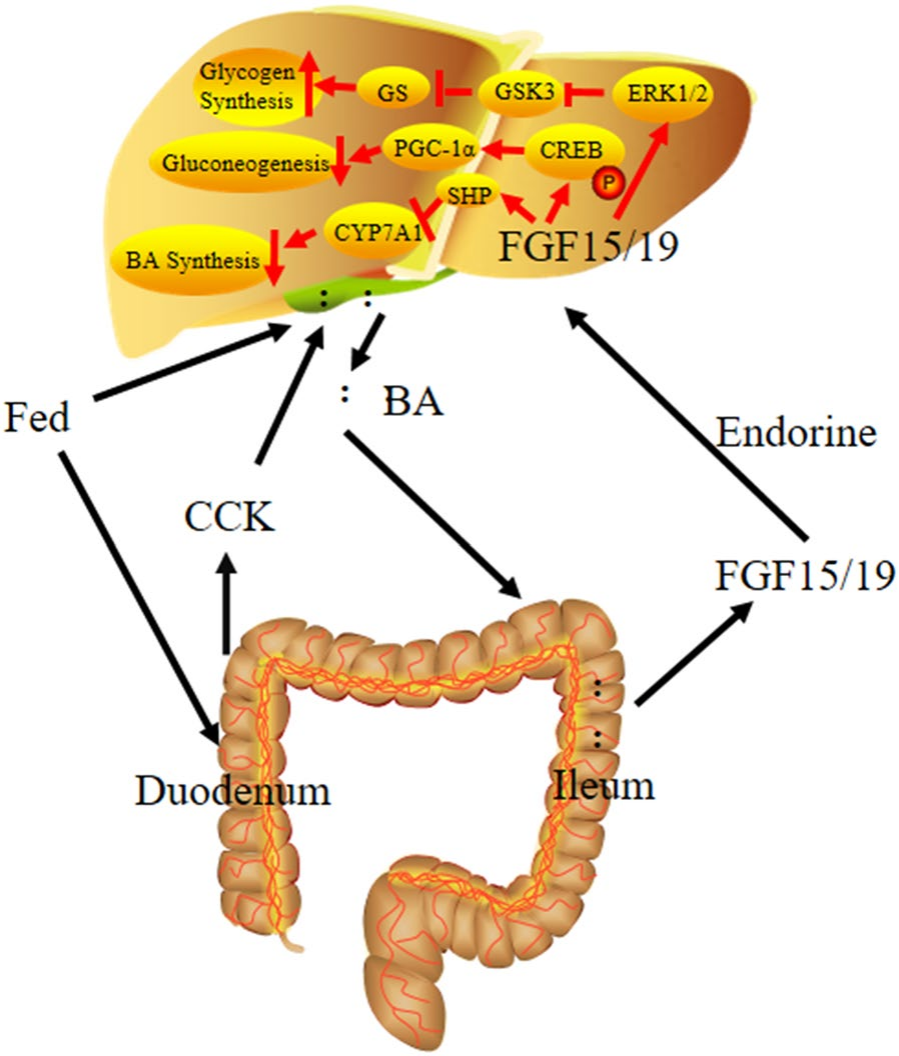
Endocrine actions of FGF15/19. In fed state, BAs stored in the gallbladder release into the intestine. FGF15/19 expression is induced in the ileum. Secreted FGF15/19 acts on FGFR/KLB receptor complexes via repress CYP7A1 decrease bile acid synthesis, to stimulate glycogen synthase (GS) activity and glycogen synthesis through inactivation of GSK3, and to repress gluconeogenesis by blocking the phosphorylation and activation of CREB

FGF15/19 is expressed in small intestine under the regulation of bile acid (BA) nuclear receptor farnesoid X receptor (FXR) [40, 41], and is a negative feedback regulator of BA synthesis and gallbladder filling (Fig. 2).

In response to fasting, BAs are stored in the gallbladder until they are needed for digestion normally [15]. In fed state, BAs are released from the gallbladder into the intestine, bind to and activate FXR, thereby induce expression of FGF19 [42]. In humans, serum FGF19 levels exhibited a rhythm with peaks occurring 90–120 min after the postprandial rise in serum BAs, and the FGF19 peaks in turn preceded the declining phase of BA synthesis [43].

In small intestine, BA induces FGF15/19 expression by activating FXR. FXR induces the expression of small heterodimer partner (SHP) in liver [44, 45]. However, unlike most nuclear receptors, SHP lacks a DNA-binding domain and binds indirectly to the CYP7A1 promoter [44–46]. Knockout studies in FXR-KO and SHP-KO mice has been demonstrated the significance of the FXR–SHP pathway in bile acid homeostasis, both of which increase CYP7A1 expression [47–49], the rate-limiting enzyme in the classical BA biosynthetic pathway [14]. FGF15/19 represses CYP7A1 by binding to the FGFR4/KLB receptor complex to activate downstream signaling cascade [43, 50]. A more recent study showed that an uncharacterized gene, Diet1, transcriptionally and post-transcriptionally influences FGF15/19 level as well as CYP7A1 level, and it co-localize with FGF19 in cultured intestinal cells [51]. This suggests that Diet1 plays a role in FGF15/19 intestine-liver axis involved in the BA synthesis. FGF15/19, a hormone made by the distal small intestine in response to BAs, also promotes relaxation and refilling of the gallbladder after a meal. Cholecystokinin (CCK) is a hormone secreted by duodenum causing gallbladder contraction to release bile, which facilitates lipid digestion. Bile acids travel to ileum, where they induce FGF15 synthesis. FGF15 in turn stimulates gallbladder filling by relaxing smooth muscle in gallbladder [15].

After a meal, besides regulation of BA synthesis and gallbladder filling, FGF15/19 has an effect on glycogen synthesis. FGF15/19 acts on FGFR/KLB receptor complexes to represses cholesterol 7a-hydroxylase (CYP7A1) through small heteromer partner [41, 52], and then increase hepatic glycogen synthase (GS) activity and glycogen synthesis in an insulin-independent manner by inducing the phosphorylation and inactivation of GSK3s [53]. Serum FGF19 levels peak approximately 3 h after a meal [43] and increase glycogen synthesis by activation of the Ras/ERK pathway; in contrast, serum insulin levels peak within 1 h after a meal and stimulate glycogen synthesis by the phosphoinositide 3-kinase (Akt) pathway [42].

In addition to glycogen synthesis, FGF15/19 also has an effect on gluconeogenesis. To date, gluconeogenesis inhibition is also differently mediated by FGF19 and insulin by dephosphorylation and inactivation of cAMP response element-binding protein (CREB) and Akt-dependent phosphorylation and FoxO1 degradation, respectively [54]. Unlike insulin, FGF15/19 represses gluconeogenesis gene expressions by promoting protein kinase B(Akt) dependent FOXO1 phosphorylation and dephosphorylation, FGF15/19 cannot activate the PI3K/Akt pathway [42]. The mechanism by which FGF15/19 blocks the expression of gluconeogenesis genes involves dephosphorylation and inactivation of the transcription factor CREB [13]. This in turn down-regulates peroxisome proliferator-activated receptor-1α (PGC1α) transcription, which subsequently decreases its binding to glucose-6-phosphatase catalytic subunit gene and phosphoenolpyruvate carborykinase gene promoters [13]. Therefore, FGF15/19 inhibits gluconeogenesis via regulating the expression of genes involved in gluconeogenesis.

In fasted status, FGF19 increased the phosphorylation of ERK1 and ERK2 in liver. FGF19 induced phosphorylation of both glycogen synthase kinase (GSK) 3α and GSK3β in animals fasted overnight, which correlated with decreased phosphorylation of Ser^641^ and Ser^645^ on glycogen synthase and increased glycogen synthase activity [53]. However, the effects of FGF15/19 and insulin on metabolism in fasted status are noticeable. Although both of them can stimulate glycogen and repress gluconeogenesis, there still are important differences as insulin acts through the insulin receptor-PI3K-Akt pathway, and FGF15/19 mediates its effects through the FGFR/KLB-ERK pathway. In addition, there are significant temporal differences. In humans, insulin is released minutes after a meal, and in rodent experiments, serum insulin concentrations and its downstream Akt phosphorylation in liver peak approximately 15 min after a high-carbohydrate or high-fat diet. In contrast, FGF15 mRNA levels in ileum and downstream hepatic ERK1/2 phosphorylation peak about 1 h after feeding [13]. Likewise, FGF19 serum levels peak near 2 h after a meal [43], and accordingly, circulating FGF19 levels in humans negatively correlate with fasting glucose levels and metabolic syndrome [55–57]. Thus, FGF15/19 acts after insulin in the transition from the fed to the fasted state.

#### Regulation of FGF21 on metabolism

**Fig. 3:**
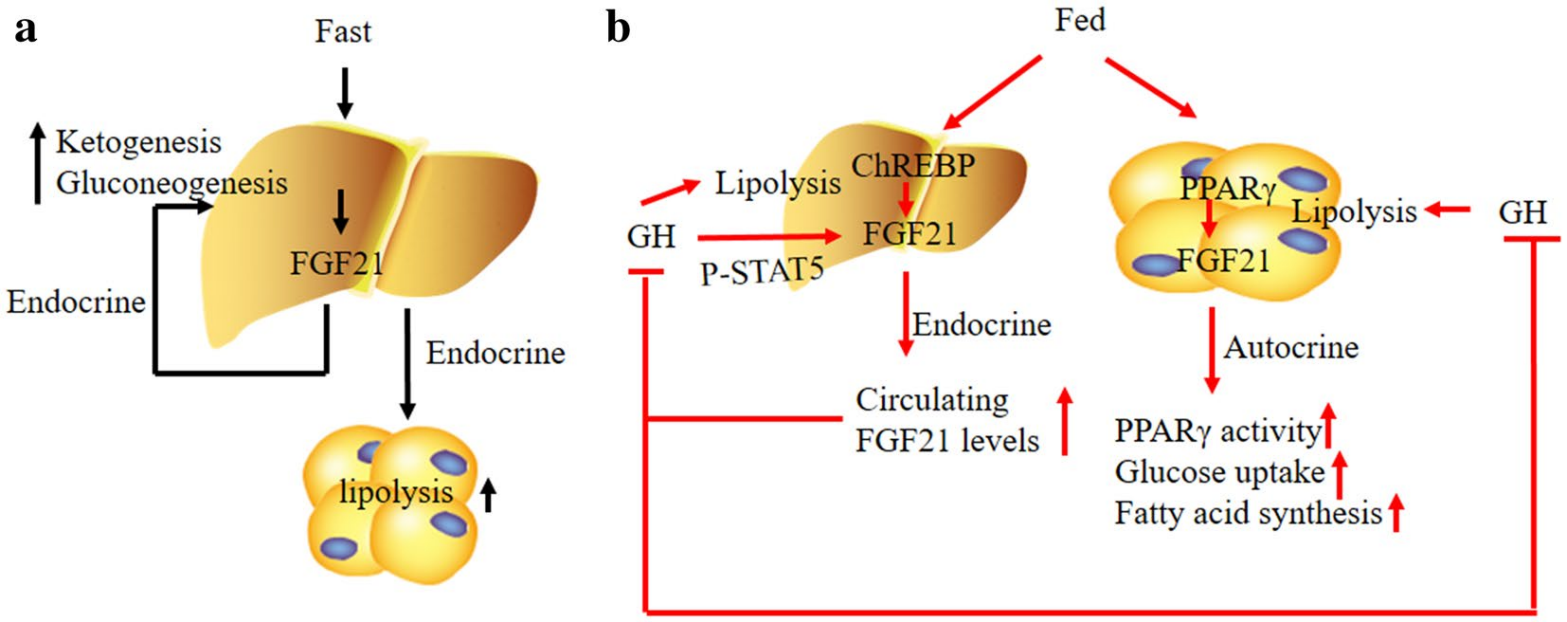
Physiology actions of FGF21. In response to fasting, FGF21 expression is induced in the liver by the PPARα. Secreted FGF21 acts as an endocrine hormone to induce ketogenesis and gluconeogenesis. In response to feeding, FGF21 expression is induced by the PPARγ in WAT and the ChREBP in liver, where FGF21 acts through an autocrine mechanism to stimulate PPARγ activity and glucose uptake and to repress lipolysis in liver via an endocrine mechanism

FGF21 is an important regulator of metabolism. A larger number of recent reports have expanded that FGF21 expression is induced in various tissues in response to fasting and feeding. The physiological function of FGF21 in the maintenance of nutritional homeostasis has been suggested (Fig. 3).

Emerging evidences have shown that fasting increases hepatic FGF21 mRNA expression and plasma FGF21 level in mice. Fasting mediated induction of FGF21 requires the peroxisome proliferator-activated receptor a (PPARα) [18, 58, 59]. PPARα can bind directly to the FGF21 gene promoter to induce its transcription [18]. It has been shown that fasting-induced FGF21 in liver increases gluconeogenesis, but does not increase glycogenolysis [60]. Gluconeogenesis is controlled by several key enzymes including fructose-1,6-bisphosphatase, glucose-6-phosphatase and phosphoenopyruvate carboxykinase. An acute FGF21 treatment leads to the gene expressions of hepatic glucose-6-phosphatase and phosphoenopyruvate carborykinase [61]. PGC-lα as a transcriptional coactivator regulating gluconeogenic gene is increased after FGF21 treatment [62]. PGC-1α knockout mice fail to induce glucose-6-phosphatase catalytic subunit and phosphoenopyruvate carboxykinase mRNA expression after FGF21 injection [60]. Taking together, PGC-1α may play an important role in FGF21 promoting hepatic gluconeogenesis. In contract to those findings, a study using mice with liver-specific deletion of PGC-1α reveals that liver PGC-1α is unnecessary in the effects of FGF21 on the gluconeogenesis [61]. The difference results from those two studies can be explained by the mouse models. One is a whole-body PGC-1α knockout and the other is a liver-specific knockout. It suggests that PGC-1α expressed in tissues other than liver affects FGF21 regulatory effects on the gluconeogenesis. For example, a study has shown that FGF21 induces PGC-1α via an indirect mechanism of central nervous system [63]. So the importance of PGC-1α induction for FGF21 action remains in question [60, 61].

Ketone bodies are produced in the liver and delivered to the brain as the major source of energy during fasting period. FGF21 is a molecule regulating lipid metabolism in response to fasting [18, 19]. Increased FGF21 promotes adipose lipolysis in white adipose tissue in an endocrine manner, and increases the fatty acids transport to the liver where they are directly oxidized for energy production or utilized as a source for ketone body formation [18]. FGF21 transgenic mice have higher serum concentrations of β-hydroxybutyrate even in the fed state [18]. PPARα is responsible for coordinating lipid oxidation and ketogenesis in the liver during starvation. Although FGF21 is one of PPARα target genes, FGF21 induces ketone body production through a mechanism distinct from that previously described for PPARα. As we mentioned above, FGF21 induces ketogenesis by stimulating lipolysis, thereby increasing the supply of free fatty acids to the liver [18, 24]. Both carnitine palmitoyl transferase-1a (CPT-1a) and hydroxymethyl glutaryl-CoA synthase-2 (HMGCS2) are rate-limiting enzymes in ketogenesis [64]. Their genes are directly changed by PPARα [65]. However, FGF21 cannot regulate their gene expressions, but increases their protein levels through a posttranscriptional mechanism [18].

Mice fed a high-fat, low-carbohydrate ketogenic diet exhibit marked increases in FGF21 expression in the liver [20, 58, 66] and in white adipose tissue (WAT) by fasting–refeeding regimens [67, 68]. These responses in the liver and WAT are likely mediated by carbohydrate response element-binding protein and PPARγ, respectively [20–23]. Notably, unlike the fasting response that elicits FGF21 release from the liver into circulation, feeding induction of FGF21 in WAT do not cause a corresponding increase in circulating levels of FGF21, so FGF21 secreted by adipose tissue promotes fatty acid synthesis in adipose tissue in a paracrine or autocrine manner [68], and liver generated FGF21 promotes adipose lipolysis in white adipose tissue in an endocrine manner [18]. So FGF21 regulates lipogenesis and lipolysis by distinct modes. Recent studies found that FGF21 acts as a negative feedback signal to terminate GH-stimulated lipolysis in adipocytes and hepatocytes. In the liver, GH stimulates transcription of the FGF21 through the signal transducer and activator of transcription 5 (STAT 5) signaling pathway [69, 70].

## Conclusions and implications

FGF15/19 and FGF21 acting on the heels of metabolic regulation factors to regulate metabolism in response to nutritional status. FGF15/19 is secreted in response to feeding. FGF21 is induced in response to diverse nutrition stressors, especially fasting. Therefore, they can be considered “late-acting’’ fed- and fasted-state hormones respectively. So we conclude that FGF15/19 and FGF21 play significant roles in coordinating nutrition homeostasis under a variety of physiological conditions.

Both FGF15/19 and FGF21 have distinct physiological effects on nutrient metabolism, though they belong to the same subfamily. FGF15/19 mainly regulates gallbladder filling, bile acid synthesis, and inhibits hepatic gluconeogenesis and lipogenesis in fed state, whereas FGF21 regulates glucose uptake, glycogen synthesis, and ketogenesis in the fed/fast state. Interestingly, it has been shown that FGF15/19 and FGF21 have potential roles in metabolic diseases, such as nonalcoholic fatty liver disease, bile acid diarrhea, cardiovascular disease and diabetes. Given the high variability in inter-individuals and interspecies, further studies are urgently needed to evaluate the legitimate therapeutic roles of FGF15/19 and FGF21 in malnutrition associated diseases.

## Abbreviations

FGF: fibroblast growth factor
GFGR: fibroblast growth factor receptor
KL: Klotho
KLB: βKlotho
GS: glycogen synthase
GSK: glycogen synthase kinase
CYP7A1: cholesterol 7a-hydroxylase
Akt: protein kinase B
PI3K: phosphatidylinositol 3-kinase
CREB: cAMP response element-binding protein
PGC-1α: peroxisome proliferator-activated receptor-1α
FXR: farnesoid X receptor
Ig: immunoglogulin
ERK: extracellular regulated protein kinases
CCK: cholecystokinin
STAT 5: signal transducer and activator of transcription 5
SHP: small heterodimer partner
PPARα: peroxisome proliferator-activated receptor α
GH: growth hormone
WAT: white adipose tissue
CPT-1a: carnitine palmitoyl transferase-1a
HMGCS2: hydroxymethyl glutaryl-CoA synthase-2
